# Health Behavior Theory in Popular Calorie Counting Apps: A Content Analysis

**DOI:** 10.2196/mhealth.4177

**Published:** 2016-03-02

**Authors:** Siena F Davis, Marisa A Ellsworth, Hannah E Payne, Shelby M Hall, Joshua H West, Amber L Nordhagen

**Affiliations:** ^1^ Department of Health Science Brigham Young University Provo, UT United States

**Keywords:** cell phones, mobile applications, telemedicine, weight loss, caloric restriction

## Abstract

**Background:**

Although the Health & Fitness category of the Apple App Store features hundreds of calorie counting apps, the extent to which popular calorie counting apps include health behavior theory is unknown.

**Objective:**

This study evaluates the presence of health behavior theory in calorie counting apps.

**Methods:**

Data for this study came from an extensive content analysis of the 10 most popular calorie counting apps in the Health & Fitness category of the Apple App Store.

**Results:**

Each app was given a theory score to reflect the extent to which health behavior theory was integrated into the app. The highest possible score was 60. Out of the 10 apps evaluated, My Diet Coach obtained the highest theory score of 15. MapMyFitness and Yumget received the lowest scores of 0. The average theory score among the apps was 5.6.

**Conclusions:**

Most of the calorie counting apps in the sample contained minimal health behavior theory.

## Introduction

The Health & Fitness category of the Apple App Store features hundreds of calorie counting apps [1]. According to a survey by the Pew Research Center, 31% of health app users track their diet using apps [2]. Integrating health behavior theory into apps has been identified as a way to increase the likelihood for long-term dietary changes in behavior [3]; however, diet-related health apps are generally void of health behavior theory [3,4].

To date, no research has analyzed the extent to which popular calorie counting apps include health behavior theory. One important limitation of previous research on general health and fitness apps is the short amount of time and engagement with apps during content analysis. Methodologies used in previous studies are useful for providing a general overview of content, but their limited scope makes it challenging to identify all of the instances of health behavior theory integration. The purpose of this study was to conduct an extensive content analysis of the 10 most popular calorie counting apps from the Health & Fitness category of the App Store. Specifically, the purpose of this analysis was to evaluate the presence of health behavior theory in the selected calorie counting apps when used extensively over the course of one week.

## Methods

### Study Design

This study design featured a content analysis of calorie counting apps available through the App Store. Two Master of Public Health graduate students trained in health behavior theory coded the apps to determine the extent to which health behavior theory constructs were present in the apps.

### Sample

iOS apps were selected because they have been identified as scoring slightly higher than Android apps on measures of user reviews and rankings [5]. Since more than half of cell phone users only download apps that are free, the sample was limited to free apps [6]. An approach similar to what has been done in previous studies was used to identify the most relevant and popular apps [4,7]. Keywords *calorie counter* and *diet tracker* were used to identify apps. The initial search returned 319 unique apps. The study sample comprised the 10 most popular apps as determined by the number of stars and reviews. The sample was limited to 10 apps to allow the graduate student coders a minimum of one week to engage with and code each app.

### Procedure

Coders each downloaded 5 different study apps to iPhones. They used a single app exclusively to track calories for all meals over the course of at least 7 days and repeated this process until all 10 apps had been used. The interface of each app was thoroughly explored and coded based on a rubric adapted from West et al and Doshi et al [3,8].

To determine the level of interrater reliability between the two graduate student coders, they each coded two preliminary apps, which entailed coding 60 theoretical items each for a total of 120 items. The researchers then calculated the kappa for interrater agreement as .809. This coefficient shows substantial agreement based on the range .61 to .80 recommended by Landis and Koch [9].

### Measurement

The measurement, including instrument selection and methodology, was adapted from a study conducted by West et al to evaluate health theory in dieting apps, which are distinct from calorie counting apps [3]. Constructs from the health belief model, transtheoretical model, theory of planned behavior, and social cognitive theory were addressed in the rubric. The coding instrument included 12 constructs relative to calorie counting (Table 1). Each of the 12 constructs was assessed on 5 levels of user interaction, leading to 60 theory-based items. The 5 levels of user interaction as described by West et al were general information or guidelines, assessment, feedback, general assistance, and individually tailored assistance [3,8].

**Table 1:** Theory integration in selected calorie counting apps (n=10).

Behavior constructs	General information^a^	Assessment^b^	Feedback^c^	General assistance^d^	Individually-tailored assistance^e^
Knowledge^f,g,h,i^	3	0	0	0	0
Perceived benefits^f,g,h,i^	1	0	0	1	0
Perceived barrier^f,g,i^	0	0	0	0	0
Perceived risks^f,h^	1	0	0	0	1
Self-efficacy^g,h,i^	1	0	0	2	1
Social norms^g,h^	0	0	0	1	1
Self-monitoring^g,i^	4	1	1	4	2
Goal setting^i^	2	3	3	2	3
Stimulus control^g,i^	2	0	0	1	4
Self-reward^g,i^	1	0	0	0	1
Social support^g,i^	2	0	0	2	5
Vicarious learning^g,i^	0	0	0	0	0

^a^App provided primarily general information or data that were not individualized.

^b^Assessment: app asked the user for current behavioral practices or strategies.

^c^Feedback: app offered comments on the user’s current behavioral practices or strategies.

^d^General assistance: app offered nonindividualized suggestions about how to change or apply a strategy (not based on assessment or feedback).

^e^Individually tailored assistance: app provided suggestions on how to change or apply a strategy specifically tailored to the user.

^f^Health belief model.

^g^Transtheoretical model.

^h^Theory of planned behavior.

^i^Social cognitive theory.

### Analysis

STATA version 13 statistical software (StataCorp) was used for analysis. Each app was coded according to the 5 levels of each of the 12 constructs. A subscale was created for each construct by summing the values of the user interaction levels. The possible range was 0-5. Next, a total theory score was assigned to each app by summing the construct subscale values. The total theory scores ranged from 0 to 60.

## Results

The number of reviews for apps ranged from 24 to 2435. The number of stars for apps ranged from 4 to 5. Across all levels of interaction, the constructs that were most commonly coded for included knowledge, self-monitoring, goal setting, social support, and stimulus control. A score was assigned to each app to reflect the extent to which theory is integrated. In general, apps lack health behavior theory. My Diet Coach obtained the highest theory score of 15. MapMyFitness and Yumget received the lowest rankings with scores of 0. The average theory score was 5.6 (Table 2).

**Table 2:** Theory scores of selected calorie counting apps.

Name of application	Theory score (0-60)
My Diet Coach	15
My Diet Diary	14
MyFitnessPal	8
MyNetDiary	7
Calorie Counter, Dining Out, Food, and Exercise Tracker	6
PhotoCalorie	4
Lose It!	1
MapMyRun	1
MapMyFitness	0
Yumget	0

## Discussion

### Principal Findings

The majority of the apps in this study only minimally integrated health behavior theory. The lack of health behavior theory integration may be an early indication of the low potential for the study apps to influence behavior long term. These apps are popular among users, as noted by number of stars and reviews, but the apps may or may not be successful in changing user behavior. App developers likely have a skillset focused on the technical aspects of development with a goal to create a popular app, not to integrate health behavior theory [4]. This gap in information highlights the need for cooperation between certified health education specialists and app developers [4].

Knowledge was only dealt with on a superficial level; the apps provided general information but did not assess the user’s knowledge in an effort to change it. In some respects, this mirrors attempts to change behavior using traditional mediums characterized by one-way transfers of information. Evidence of self-monitoring was identified in study apps, but this would be expected because the purpose of calorie counting apps is to track diet.

Including theoretical health constructs such as goal setting and social support in the creation of apps is a progressive step for developers, but future research is needed to determine the effectiveness of these constructs to change health behavior. Additional studies should measure how effective these goals are—for example, whether goals are considered SMART goals (specific, measurable, achievable, realistic, timely). Social support received the single highest score; many apps provided options to share successes in calorie counting and weight loss on different social networking sites.

There were encouraging examples of health behavior theory integration into study apps, but there were also some constructs that were missing and these could be easily integrated into future versions. Self-efficacy is defined as one’s belief in his or her ability to produce a desired result. For the purposes of this study, self-efficacy could be measured by users’ confidence in their ability to eat fewer calories. A high self-efficacy enhances human accomplishment by promoting a strong assurance in the ability to master difficult tasks [10]. Weight loss can be a daunting feat to accomplish, and low self-efficacy is a barrier to improving diet and achieving a healthy weight. Future apps can address increasing self-efficacy by incorporating a confidence rating scale for users to more easily conceptualize improvements in self-confidence. In addition, future apps can implement individually tailored messages of support and encouragement to boost user confidence.

According to McAlister et al, vicarious learning is “learning to perform new behaviors by exposure to interpersonal or media displays of them, particularly through peer modeling” [11]. Examples of successful dieting behavior can influence the behavior of people who are trying to diet. Future apps could include videos of people successfully counting calories in public settings or links to social media to foster peer support activities. These interactions could help the person dieting learn from positive behaviors and make healthier decisions.

Self-reward has been defined as “short-term and frequent rewards that people give themselves” [11]. Individuals are able to feel satisfaction in their progress as they provide themselves with these rewards [11]. Self-reward can motivate the individual to press forward in dieting and calorie counting when results are not immediate. Many times, individuals are capable of enduring short-term negative effects acknowledging that they can lead to positive long-term outcomes [11]. An example of self-reward in an app would be to encourage the user to set aside money every day they count calories in order to buy things that bring fulfillment. Another example of how an app could integrate this construct would be sending an individualized email to remind users to reward themselves for counting calories. This reward may be set by the individual in compliance with their desires and needs.

### Limitations

Several limitations should be considered when interpreting the results of this study. First, the researchers limited their sample to 10 calorie counting apps. It is possible that an evaluation including a larger sample size would have produced different results. However, previous studies using larger sample sizes have also found that health apps lack health behavior theory [3,4]. A small sample size was selected for this study to enable coders to use each app for an entire week to evaluate the presence of health behavior theory. Second, apps that included physical activity components were included in the sample. It is possible that some of the theories observed in the apps were intended for physical activity and not calorie counting. Consequently, the results may overestimate the presence of health behavior theory. Apps that contained a physical activity component were included in the sample because the majority of calorie counting apps measure both calorie intake and calorie expenditure, and calorie expenditure is measured using physical activity.

### Conclusion

The majority of apps available today allow users to acquire knowledge and track behavior. While this is a step in the right direction, it is not sufficient for behavior change. Future apps should incorporate constructs such as self-efficacy, vicarious learning, and self-reward to increase positive outcomes and behavior change in users.
